# Sociodemographic and Health Behaviour of Frequent, Avoidable Emergency Department Users in Ontario, Canada: A Population-based Descriptive Study

**DOI:** 10.5811/westjem.46551

**Published:** 2025-10-21

**Authors:** Cameron Thompson, Tristan Watson, Michael J Schull, Jessica Gronsbell, Laura CA Rosella

**Affiliations:** * University of Toronto, Dalla Lana School of Public Health, Toronto, Ontario, Canada; † Sinai Health, Schwartz/Reisman Emergency Medicine Institute, Toronto, Ontario, Canada; ‡ ICES, Toronto, Ontario, Canada; § University of Toronto, Dalla Lana School of Public Health, Institute of Health Policy, Management & Evaluation, Toronto, Ontario, Canada; ¶ University of Toronto, Department of Statistical Sciences, Toronto, Ontario, Canada; || Sunnybrook Research Institute, Sunnybrook Health Sciences Centre, Toronto, Ontario, Canada; # University of Toronto, Department of Medicine, Toronto, Ontario, Canada; ** University of Toronto, Department of Computer Science, Toronto, Ontario, Canada; †† University of Toronto, Department of Family and Community Medicine, Toronto, Ontario, Canada; §§ Trillium Health Partners, Institute for Better Health, Mississauga, Ontario, Canada; |||| University of Toronto, Temerty Faculty of Medicine, Toronto, Ontario, Canada

## Abstract

**Introduction:**

Frequent users are a small but important group of patients in the emergency department (ED). This group is often the target of interventions that redirect visits to other areas of the healthcare system under the premise that some of these visits could be best managed elsewhere. Most existing interventions do not consider sociodemographic factors when targeting specific populations, while larger scale policy initiatives often do not reach those who would most benefit from alternative points of healthcare access. In this study we use population-level survey data linked to health administrative data to describe frequent ED users and those whose visits are potentially avoidable and could benefit from additional points of healthcare access.

**Methods:**

This was a population-based cohort study of responses from 18–74 year-old Ontario residents to the Canadian Community Health Survey from 2001–2014, which we linked to administrative health data for one-year following survey completion. We categorized participants according to the frequency of their ED use in the year following survey date and whether any of their visits were potentially avoidable. Associations between category of ED use and various sociodemographic, health, and behavioural factors were examined with multinomial logistic regression.

**Results:**

A total of 181,369 eligible respondents were included in this study. Of these, 1,460 (0.8%) were frequent users (four or more visits) with one or more potentially avoidable visits in the year following survey date. Compared to non-ED users, frequent users with avoidable visits were associated with the lowest quintile of household income (aOR: 1.91, 95% CI: 1.37, 2.65), rural-dwelling (aOR: 1.44, 95% CI: 1.18, 1.77), and the highest quintile of material resource deprived neighbourhoods (aOR: 2.23, 95% CI: 1.47, 3.36). They were more likely to have poor self-reported physical (17.2% vs 9.0%) and mental health (4.1% vs 2.7%) compared to total cohort, and more likely to have comorbidities (63.3% vs 48.7%), but less likely to access a usual provider of care for their healthcare needs (33.3% vs 28.2% without a usual provider of care).

**Conclusion:**

This study provides a novel description of frequent ED users for whom some of their visits were potentially avoidable. As efforts are made to redesign access to primary and community care, and with increasing emphasis on virtual care and other initiatives to reduce avoidable ED use, the healthcare system should ensure that these interventions are responsive to the needs of the people at higher likelihood of needing them.

## INTRODUCTION

Frequent users represent a small but important group of patients in the emergency department (ED). Although no standard definition exists, they are often defined as those with four or more visits within a year.^1^,^2^ These individuals represent less than 5% of visitors but account for up to 30% of all ED visits.^1^–^6^ Frequent users have been the focus of many initiatives to reduce frequent ED use in favour of other options for care, both in the form of large-scale policy (eg, telehealth, ED dis-incentive programs) and smaller scale interventions (eg, case management programs, individual care plans), which have had mixed results in curtailing frequent ED usage.^5^–^9^ A recent mapping review by Memedovich et al identified 58 studies from 2013–2023 that aimed to address frequent users in an ED setting.^7^ Most existing programs were created for specific conditions or for populations such as older adults, but the authors identified that very few of the studies considered mental health conditions or sociodemographic characteristics, such as income and racial or indigenous identity.^7^

In Canada, there has been an increase in health system strategies, including team-based primary care and digital health solutions such as virtual care and telehealth, which offer differing approaches to providing alternative points of care for patients who may not require management in the ED setting. In Ontario, patients receiving primary care delivered by a team-based model had lower rates of ED use compared to patients participating in nonteam practice.^10^ However, these team-based models are not universally available— particularly for patients living in more rural or remote regions who may be in greater need of healthcare.^10^,^11^ Virtual care in various forms proliferated throughout the COVID-19 pandemic as a means to reduce in-person healthcare use, including ED visits. However, when delivered by clinicians without an ongoing relationship with the patient (ie, emergency or walk-in services), virtual care was not found to necessarily keep patients from visiting the ED.^12^,^13^ It is also not clear whether virtual care is reaching those most in need or even the most appropriate care modality for many conditions. Virtual care evaluations in both Ontario and British Columbia revealed that services were primarily accessed by individuals who already had primary care access, were educated and middle-aged, and lived closer to urban centres.^12^,^14^,^15^

As health systems across many jurisdictions continue to move towards team-based primary care models, and as virtual care and other digital health solutions proliferate, it is important to understand the patient populations who could most benefit from alternative access points to healthcare. Given the heterogeneous nature of frequent or avoidable ED users, these health-seeking behaviours may not have a singular root cause but may be made up of multifaceted, upstream factors including perceived urgency of the acute health issue, beliefs, and timeliness of other forms of healthcare.^1^,^2^,^16^,^17^ To design policy and interventions in the most effective way and target those with the greatest need, we need a comprehensive understanding of the social determinants of frequent ED use and of the frequent ED users for whom their visits may be amenable to other areas of care, as well as those who will continue to rely on the ED as their primary point of healthcare access.^17^

**Population Health Research Capsule**

What do we already know about this issue?

*Frequent users of the emergency department are a diverse group, and existing ED diversion initiatives often do not consider social factors in their implementation*.

What was the research question?

*We sought to describe the sociodemographic and behavioural characteristics of frequent, avoidable ED users*.

What was the major finding of the study?

*Frequent ED use in the year prior was strongly associated with frequent, avoidable ED use (OR 82.13, 95% CI 61.60–109.50)*.

How does this improve population health?

*Social factors must be considered in population-level ED diversion programs to provide contextually appropriate in-ED programs to manage frequent ED users*.

Existing studies describing individuals who use the ED frequently or for potentially avoidable reasons have typically relied on either electronic health record data or large-scale administrative data studies that lack the social, demographic and broader determinants of health data available in more detailed health surveys.^16^–^19^ In this study we used a population-representative survey of community-dwelling Canadian adults linked to future healthcare-use data to provide a more detailed characterization of frequent ED users and those whose visits are potentially avoidable. This was accomplished through the application of the Andersen-Newman Behavioural Model (ANBM)^20^–^22^ for healthcare use and the categorization of sociodemographic, health behaviour, health status, and geographic variables with known or theoretical associations with frequent or avoidable ED use into predisposing, enabling, or need factors based on their theorized mechanism of action on ED use. We hypothesized that frequent, avoidable ED use would be associated with each of predisposing, enabling, and need factors.

## METHODS

### Data Sources

Ontario respondents to the Canadian Community Health Survey (CCHS) between 2001–2014 were linked to population-based health administrative data. The CCHS is a cross-sectional survey administered annually or bi-annually by Statistics Canada, which through the use of multistage sampling consisting of stratified cluster sampling and random digit dialing and the application of sampling weights, is considered representative of community-dwelling Canadians over 12 years of age.^23^ The CCHS methodology has been described in detail elsewhere.^23^ Participants may consent to the linkage of their survey response with administrative health data. For this study, respondents were only included if they were between the ages of 18–74 years at the time of interview, and respondents were excluded if they had previously completed the survey during the study period. The CCHS contains data on household income, demographics, health behaviours (e.g., alcohol consumption, physical activity), and health status (e.g., self-perceived physical health, mental health).^24^

The CCHS has been used extensively in previous studies of health system use or performance.^25^,^26^ These datasets were linked using unique encoded identifiers and analyzed at ICES (formerly the Institute for Clinical Evaluative Sciences, which is now identified solely by the initialism ICES). Data on most publicly funded healthcare use in the province are housed in ICES’ various databases, given universal healthcare coverage.^27^ The Ntional Ambulatory Care Reporting System (NACRS) database contains mandatory reporting of all hospital-based and community-based ED and ambulatory care visits for individuals with an Ontario Health Insurance Plan number.

The NACRS dataset provided information regarding each individual visit before and in the year following the CCHS interview, including date and time, length of stay, presenting complaint (Canadian Emergency Department Information System [CEDIS]),^28^ discharge diagnosis (*International Classification of Diseases* Revisions 9 or 10 codes), mode of arrival, and acuity.^29^ The Ontario Marginalization Index contains dissemination-area and census-tract level measurements including types of residential density and family structure characteristics (households and dwellings), access to basic material needs (material resources), and ratio of seniors and children to working age population (age and labour force).^30^ We calculated Johns Hopkins ACG ^®^ System Aggregated Diagnosis Groups (ADG) using a two-year lookback to reflect five clinical dimensions: duration of the condition; severity of the condition; diagnostic certainty; etiology of the condition; and specialty care involvement.^31^,^32^

Visits to the ED were categorized as avoidable based on two definitions previously used in Canadian administrative data research^33^: ambulatory care sensitive conditions (ACSC)^34^ and sentinel nonurgent conditions (SNC).^35^ The ACSC are typically chronic conditions (e.g., asthma, congestive heart failure) for which effective ongoing care could reduce the risk of acute illness. They are commonly used for regular health system monitoring in Canada,^33^,^34^ while SNCs are low-acuity conditions (e.g., upper respiratory tract infections) that could be managed in alternative, primary care settings.^33^,^35^ These two definitions were chosen to reflect different pathways through which avoidable ED visits can occur, while also being more specific than broad definitions based solely on acuity.^33^ Complete criteria for both definitions are presented in Supplemental File 1. Respondents were categorized based on frequency of ED usage one-year following CCHS interview (four or more visits, one to three visits, zero visits) and whether any of their visits were potentially avoidable (one or more vs no avoidable visits), resulting in five categories of ED usage. Categories of ED use were as follows: frequent ED use (≥ 4 visits) with at least one avoidable visit; frequent ED use without avoidable visits; infrequent ED use (1–3 visits) with at least one avoidable visit; infrequent ED use without avoidable visits; and no ED utilization.

Covariate selection was guided by previous literature on frequent ED use, avoidable ED use, or health services utilization and categorized according to the ANBM.^20^–^22^ The ANBM had been used previously to describe non-emergent ED use in non-Canadian settings.^36^ Covariates were categorized as either predisposing factors, enabling factors, or need factors influencing frequent, avoidable ED use. Predisposing factors were variables inherent to the individual, predominately sociodemographic variables such as age, sex, or ethnicity. Enabling factors were variables which may influence an individual’s ability to access healthcare or the way in which they access healthcare such as having a regular family doctor. Need factors were variables associated with acute or ongoing medical need, such as self-reported health or comorbidities.^20^–^22^ A complete list of covariates included in this study is included in Supplemental File 2.

### Statistical Analyses

Covariates with missing data were imputed using 10-times multiple imputation. Variables with greater than 5% missingness included first language (27.7%), life satisfaction (15.6%), self-reported mental health (15.5%), and income (5.9%), the former three of which were not asked in one or more cycles of the CCHS (2001 for life satisfaction and self-reported mental health, 2011/12 and 2013/14 for first language). We fit logistic regression models for categorical variables and used predictive mean matching for continuous variables containing all other covariates included in this analysis to inform imputation. Distributions of data were examined before and after imputation to ensure consistency. All analyses were performed using the 10-times imputed data.

We calculated descriptive statistics in the form of weighted frequency distributions for all covariates, both across the total cohort and stratified by category of ED use and weighted using CCHS sampling weights. To assess associations between individual covariates and ED use, we performed multinomial logistic regression models using category of ED utilization as the nominal outcome with ‘no ED utilization’ as the reference outcome. We assessed relationships between covariates and the outcome through unadjusted models, age-adjusted models, and models adjusting for demographics, behavioural factors, comorbidities, and past healthcare use (a *fully-adjusted* model). Multicollinearity was assessed using variance inflation factors. We performed both multiple imputation and all statistical analyses using SAS v9.3 (SAS Institute, Cary, NC).

### Ethics Approval

This study received research ethics approval from the University of Toronto Research Ethics Board (Human Protocol Number: 44462).

## RESULTS

After exclusions, a total of 181,369 (77.5%) individuals who consented to linkage were included in this study. When applying the criteria for ED utilization, we identified 1,460 participants (0.8%) as having four or more ED visits within the following year (frequent) with one or more visits being considered avoidable. A further 3,062 participants (1.7%) were categorized as frequent users but with no identified avoidable visits, while 5,245 participants (2.9%) had fewer than four visits but with one or more avoidable, and 35,313 (19.5%) with one to three ED visits and no identified avoidable visits in the year following survey completion. All other CCHS participants had no ED visits within Ontario in the year following survey completion. The complete weighted distribution of all covariates in the cohort and stratified by ED use category is in Table 1, and weighted multinomial logistic regression is in Table 2.

### Predisposing Factors

There was a higher proportion of older individuals (61–74 years of age) in the frequent, avoidable ED user group than in the overall CCHS population. In the fully adjusted model, female sex was associated with both frequent, avoidable ED use (adjusted odds ratio [AOR] 1.35, 95% CI 1.09–1.66) and infrequent, avoidable ED use (AOR 1.50, 95% CI 1.35–1.66) compared to male sex. Frequent, avoidable ED users were both less likely to have a post-secondary education (AOR 0.57, 95% CI 0.45, 0.72) and more likely to be in the lowest quintile of household income (AOR 1.91, 95% CI 1.37–2.65) compared to those who did not have an ED visit within the year following survey completion. Frequent, avoidable users were less likely to be married or in a common-law relationship than infrequent or non-ED users. Individuals in the frequent, avoidable ED user group reported higher levels of life stress and lower levels of overall life satisfaction than other groups; however, after adjustment both factors were also not statistically significant.

### Enabling Factors

Neighbourhood-level material resource deprivation (the inability of individuals and communities to attain their basic material needs)^30^ was more common amongst individuals with frequent, avoidable ED use, with 30.4% living in the highest quintile of most-deprived neighbourhoods compared to 18.4% of individuals with no ED use. Neighbourhood-level household and dwelling stability was similarly associated with ED use, as individuals living in the most residentially stable neighbourhoods were least represented amongst the frequent, avoidable ED users (8.0%) compared to 21.3% of individuals with no ED use living within the same neighbourhood quintile. Neighbourhood-level proportion of population outside working age (age and labour force) was also associated with frequent ED utilization, as 35.0% of frequent, avoidable ED users resided in neighbourhoods in the highest quintile of dependency. All neighbourhood-level marginalization metrics were associated with each level of ED use; however, effect sizes on unadjusted, age-adjusted, and fully adjusted models are highest in the frequent, avoidable ED-user and frequent ED-user categories.[Fig f1-wjem-26-1622]

Frequent, avoidable ED use was more likely to be by White (91.3%) and Canadian born (83.7%) individuals than their non-avoidable frequent and infrequent counterparts. This association remained with adjustment for other covariates, as both visible minorities and immigrants who had been in Canada for longer than 10 years were at reduced likelihood of being in the avoidable ED use categories compared to White or Canadian-born individuals.

All ED use categories had a similar proportion of individuals who reported a connection to a regular family doctor in the CCHS survey. However, after adjusting for other covariates, having a regular family doctor was associated with a reduced likelihood of frequent, avoidable ED usage (AOR 0.73, 95% CI 0.56–0.95) and infrequent, avoidable ED usage (AOR 0.82, 95% CI 0.69–0.97). In both the frequent ED use categories, a greater proportion of participants were identified as having more than three ambulatory care healthcare encounters without a usual provider of care (UPC) in the 18 months prior to survey completion (33.7% and 34.7%, respectively) compared to their infrequent ED user counterparts.

### Need Factors

Individuals in the frequent, avoidable ED-use category reported or were associated with poor health through several metrics: 17.2% of participants in the frequent, avoidable ED-user category self-reported poor general health compared to 5.4% of individuals with infrequent, non-avoidable ED use. Self-reported mental health was also more commonly poor (4.1%) or fair (11.3%) in the frequent, avoidable ED-use category compared to infrequent, non-avoidable ED users (2.1% poor and 6.5% fair, respectively). Frequent, avoidable ED use was also more likely by. individuals who had comorbidities at the time of the interview, with 63.3% of this category having one or more identified chronic conditions compared to 48.7% of the overall, weighted cohort.

Lastly, past ED use of any kind was strongly associated with the category of ED use. Nearly one-third (32.8%) of individuals in the frequent, avoidable use category were frequent users the year prior, and this association with frequent, avoidable use persisted after adjustment for other covariates (AOR 82.13, 95% CI 61.60–109.50).

## DISCUSSION

In this analysis of a population-based survey linked to administrative health data, we examined the predisposing, enabling, and need factors associated with frequent and avoidable ED users in Ontario. Our study provides a novel description of not just clinical factors, but also sociodemographic, behavioural, and geographic factors which are associated with frequent, avoidable ED use.

Based on our composite definition of avoidable^33^ and commonly used benchmark for frequent use,^1^ we identified a relatively small cohort of community-dwelling Ontarians (0.8%) who both make a large number of ED visits and for whom at least some of these visits may be best managed in areas of the healthcare system outside the ED. This category of ED users is generally younger, less educated, and of a lower household income quintile. They more often live rurally, and in neighbourhoods that have higher levels of material resource deprivation and lower levels of residential stability. They were also more commonly English-speaking and Canadian born, which is consistent with previous literature on Canadian immigration and ED use.^37^ Intervening on this group within or outside the ED setting could improve patient care as frequent ED users have been shown to have increased likelihood of mortality and hospital admission compared to non-frequent ED users.^38^

While this study identifies a number of predisposing and enabling factors associated with frequent, avoidable ED use, previous studies applying the ANBM or similar behavioural models have identified need as the largest driver of non-urgent or non-emergent ED presentations.^36^,^39^ This study also demonstrated a strong association between need and frequent, avoidable ED use as self-perceived health, as well as administratively identified chronic conditions and ADG quartile were all highest in this category. However, unlike some previous studies,^36^ in this study both enabling and predisposing factors demonstrated strong associations with frequent, avoidable ED use. This may be partly attributed to the consideration of frequency of ED use in addition to avoidable, non-urgent, or non-emergent ED presentations alone. Emergency department use, both frequent and potentially avoidable, is multifactorial—associated with several predisposing, enabling, and need factors—and the results of this study highlight that.

Previous studies have shown that patients seeking care in the ED for conditions perceived as non-urgent or avoidable do so for a variety of reasons ranging from primary care unavailability to perceived urgency of their medical issue, and that this group is sociodemographically heterogeneous.^40^,^41^ Frequent, avoidable ED users in this study are also, after adjustment, less likely to self-report having a family doctor and more likely to have repeated healthcare use without that use centralized on a usual care provider. Being rostered to a primary care physician has not been shown to have a large effect on frequent or avoidable ED use based on previous studies.^42^,^43^ However, patients with high levels of continuity of care with a primary care physician have been shown to have lower rates of emergency service ED use, which may indicate the importance of having an accessible usual care provider.^44^,^45^

This study highlights the role of not just having a primary care physician (via self-report response), but of having high continuity of care with a single, usual care provider which may be an indicator of having accessible care. Even amongst patients with an existing primary care connection, sociodemographic factors, geographic factors, and barriers to access may make the ED a more palatable choice when facing a medical emergency due in part to its immediate availability and access-point to specialist care and diagnostic workup.^46^,^47^ Widespread enrollment in team-based primary care models have shown promise in reducing the impact of neighbourhood-level marginalization on low-acuity ED utilization in a Canadian setting,^48^ which has been previously shown to correlate to higher rates of ED utilization in a pediatric population.^49^

Avoidable or low-acuity ED use has long been considered a metric of poor health system performance,^50^ and this study further describes individuals and groups who may be falling through the gaps of primary care and could benefit most from more widespread primary care reforms.^51^ However, since predisposing, enabling, and need factors beyond primary care continuity also demonstrate associations with frequent, avoidable ED use, it is likely that primary care reform alone is insufficient and that many individuals will benefit from other forms of targeted ED-based interventions or community programs.

A recent review by Jeyaraman et al described the plethora of interventions designed to manage ED flow, broadly categorizing these as within the ED and outside the ED.^52^ Many of the outside-ED interventions focused on improving access to primary care and demonstrated good effectiveness; however, these largely took place in urban centres.^52^ As we have seen with the roll-out of virtual urgent care programs, large-scale outside-ED interventions to provide patients with alternatives to the traditional ED have often ended up targeting those with existing access.^14^,^15^ The characteristics of individuals characterized by frequent, avoidable ED use identified in this study differ greatly from those who have been identified as using virtual urgent care services, indicating that future similar interventions should be carefully targeted towards those with the greatest access need, specifically those living rurally, in marginalized neighbourhoods, and those with poor overall health.

Canadian recommendations for equitable and sustainable virtual urgent care include technological considerations for those living in rural areas or with limited high-speed internet access, and availability that reflects local access needs.^53^ At the same time, the strong association between previous frequent ED use and current frequent, avoidable ED use indicates the need for within-ED programs such as case management for frequent users. There is a need to continue to design ED programs for frequent users but also to ensure that EDs are equipped to handle the needs of people who heavily rely on them for healthcare. However, as highlighted by the review by Memedovich et al, there is a need to integrate the broader social determinants of health along with patterns of ED use when designing and implementing ED interventions to reduce or manage frequent use, ensuring that they are adequately designed to meet the complex needs of the patients that would benefit from them.^7^

## LIMITATIONS

Firstly, while this study uses population-based administrative health data and a survey which is considered representative of community-dwelling adults, it is limited in scope by those who are not included within the sampling frame of the CCHS.^24^ Of relevance is the lack of homeless representation, who are not included in the CCHS. People experiencing homelessness represent an important cohort of ED users, constituting as high as 19% of all ED visits in some settings,^54^ and with a high proportion of frequent users.^55^ However, the interventions and policy to improve healthcare access and reduce ED usage amongst this population is more often tied to provision of housing rather than what would be informed by the results of this study,^56^ which is more applicable to a community-dwelling population. There is a challenge of high-quality data in this population, and this should be a focus of future data collection efforts.

Secondly, the definition of “avoidable” used in this study is a composite definition based on two commonly used definitions in Canadian administrative data research.^33^ This definition is not comprehensive and will not capture all ED visits that may be managed elsewhere; however, the sentinel nonurgent condition definition in particular is more specific than other primary care-sensitive definitions,^35^,^50^ resulting in fewer avoidable categorizations and more conservative estimates. Furthermore, limitations with administrative data do not capture the nuance of patient decision-making and complex factors that go into seeking care in the ED, meaning that the visits captured in this analysis provide only a generalization of the types of visits that may be avoidable and the characteristics of patients at a population-level that make these visits, rather than an accurate count of every unique, avoidable ED visit within Ontario.

Next, survey data used in this study was from 2014 or earlier, as methodologic changes in the 2015 cycle of the CCHS led to a recommendation against pooling data from 2014 or earlier with data from 2015 to present.^57^ At the time of study completion, only 2015–2016 data was available, which has been analysed in this context as a single-cycle study without linkage to administrative health data.^58^ As subsequent CCHS cycles become available and linkable to administrative health data, we would recommend re-doing this analysis with data from 2015 to present. Survey data from the CCHS are entirely self-report and subject to response bias; the data are also cross-sectional and may not reflect the status of participants exactly at the time of their ED use. However, using the year immediately following CCHS interview as the window for ED use will mitigate this. Lastly, results of this study are specific to a Canadian population and health system and may not reflect all health systems. Nonetheless, there are many health systems in the world which are facing similar challenges, and these methods could still be applicable to other settings.

## CONCLUSION

This study provides a comprehensive description of frequent ED users for whom at least some of their visits could more appropriately be managed outside the ED. This is the first study to our knowledge to look at the intersection of frequent and avoidable ED use using administrative health data linked to population-representative health survey data for community-dwelling adults. Frequent, potentially avoidable ED use is associated with several predisposing, enabling, and need factors. Strongest associations were seen with female sex, low household income and education, those living rurally residing in a neighbourhood with high material deprivation and low residential stability, and poor continuity of primary care, along with poor, self-reported health and presence of one or more comorbidities. Similarly, this study also describes those characterized by frequent, avoidable ED use as having been frequent ED users in the prior year as well, indicating a need for ongoing within-ED programs to ensure EDs are well-equipped to meet the needs of these patients. As efforts are being made to redesign access to primary and community care, such as with Ontario Health Teams, and with increasing emphasis on virtual care and other initiatives to reduce avoidable ED utilization, our study shows that consideration should be made to ensure that these interventions are accessible and respond to the needs of the people at higher risk of needing them, including rural populations and those of low socioeconomic status.

## Supplementary Information





## Figures and Tables

**Figure 1 f1-wjem-26-1622:**
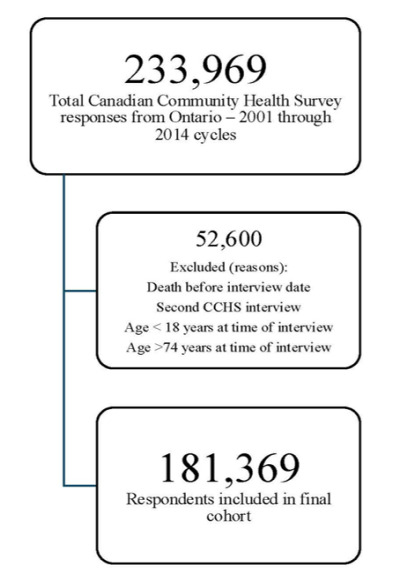
Flow diagram of inclusion into final cohort from all 2001–2014 Canadian Community Health Survey respondents in a study of frequent emergency department users. *CCHS*, Canadian Community Health Survey.

**Table 1:** The weighted distribution of predisposing, enabling, and need factors, across ED usage categories, for adult Ontarians from the 2001–2014 Canadian Community Health Survey cohorts.

Characteristic	Total	Frequent (≥ 4 visit), avoidable ED user	Frequent (≥ 4 visit), non-avoidable ED user	Infrequent (1–3 visit), avoidable ED user	Infrequent (1–3 visit), non-avoidable ED user	No ED usage
	N = 181,369	n = 1,460	n = 3,062	n = 5,245	n = 35,313	n = 136,289
Predisposing Factors						
Age group						
18 – 30	26.9	27.3	29.9	26.7	27.1	23.6
31 – 40	17.7	14.6	15.3	18.9	19.3	20.2
41 – 50	18.2	13.1	19.2	16.6	19.9	22.3
51 – 60	17.4	18.2	16.0	17.8	16.6	18.3
61 – 74	19.8	26.8	19.6	20.0	17.1	15.5
Sex (male)	44.4	37.2	47.0	38.5	49.6	49.7
Education level						
Less than secondary	19.5	30.0	22.9	17.5	15.8	11.3
Secondary graduate	20.5	19.4	21.9	20.5	21.0	19.8
Some post-secondary	8.4	9.3	8.2	8.0	8.7	7.8
Post-secondary certificate	51.6	41.3	47.0	53.9	54.5	61.2
Household income						
Q1 (lowest)	20.7	31.1	25.8	15.6	17.3	13.6
Q2	16.9	17.4	19.0	16.4	16.4	15.3
Q3	20.1	21.2	19.3	21.8	19.2	18.9
Q4	21.8	18.0	19.6	23.6	23.3	24.7
Q5 (highest)	20.5	12.2	16.4	22.6	23.7	27.6
Marital status						
Married or common law	59.4	54.8	53.4	61.5	61.7	65.5
Other	40.6	45.2	46.6	38.5	38.3	34.5
Cigarette-smoking status						
Non-smoker	46.4	40.1	39.1	45.5	49.8	57.2
Heavy smoker (≥ 1 pack/day)	5.9	6.3	8.5	5.7	5.6	3.3
Light smoker (< 1 pack/day)	24.3	28.8	30.3	22.5	22.1	17.9
Former smoker	23.5	24.8	22.1	26.3	22.5	21.6
Alcohol consumption (prior year)						
Non-drinker	64.1	75.0	67.1	63.5	58.7	56.1
Light or moderate drinker	27.3	17.3	23.9	27.9	31.6	35.9
Heavy drinker	8.6	7.7	9.0	8.6	9.7	8.0
Physical activity						
Very active	25.0	22.4	24.6	25.3	26.6	26.1
Moderately active	23.5	25.9	18.2	24.3	23.7	25.1
Inactive	51.5	51.7	57.1	50.4	49.7	48.8
Life stress						
Low (a bit, not very, none)	71.0	67.5	65.9	73.2	72.3	76.3
High (quite a bit, extreme)	29.0	32.5	34.1	26.8	27.7	23.7
Life satisfaction						
Very high	33.5	31.9	26.3	37.6	34.7	37.2
High	48.7	44.4	49.0	49.1	49.5	51.5
Neutral	12.5	15.0	16.2	10.4	11.6	9.0
Low	4.0	6.6	6.4	2.3	3.1	1.8
Very low	1.3	2.0	2.1	0.7	1.1	0.5
Body Mass Index						
Underweight, <18.5	6.3	8.2	7.6	5.5	5.6	4.7
Normal weight, 18.5–24.9	32.8	26.0	34.1	31.6	34.2	38.3
Overweight, 25–29.9	31.6	29.3	27.2	31.9	33.9	35.4
Obese, > 30	29.3	36.5	31.1	31.0	26.2	21.6
Enabling Factors						
Neighbourhood-level material resources						
Q1 (least deprived)	14.9	10.5	11.2	13.5	17.6	21.5
Q2	18.6	15.9	16.7	20.0	19.3	21.4
Q3	19.3	17.6	18.7	20.9	19.8	19.8
Q4	22.3	25.6	24.3	22.4	20.3	19.0
Q5 (most deprived)	24.9	30.4	29.2	23.2	23.1	18.4
Neighbourhood-level household and dwelling						
Q1 (least unstable)	14.7	8.0	13.6	13.6	17.1	21.3
Q2	19.0	17.0	18.1	20.5	19.2	20.2
Q3	20.9	23.6	19.2	23.5	19.9	18.5
Q4	22.7	28.7	22.1	22.4	21.1	19.4
Q5 (most unstable)	22.6	22.7	27.0	20.0	22.6	20.6
Neighbourhood-level age and labour force						
Q1 (least dependency)	17.3	11.5	17.4	14.7	19.8	23.1
Q2	19.1	15.1	18.6	18.0	21.6	22.3
Q3	18.4	14.6	18.3	19.7	19.3	19.9
Q4	21.1	23.8	21.5	23.1	19.4	18.0
Q5 (most dependency)	24.1	35.0	24.4	24.5	19.9	16.7
Racial or ethnic origin						
Visible minority	15.4	8.7	16.2	10.0	18.0	24.4
White	84.6	91.3	83.8	90.0	82.0	75.6
First language						
Other	26.4	23.4	26.3	21.0	27.8	33.5
English	73.6	76.6	73.7	79.0	72.2	66.5
Immigration status						
Canadian-born	75.9	83.7	75.3	81.7	72.9	65.9
Immigrant, < 10 years	5.1	3.1	3.7	3.7	6.1	9.0
Immigrant, ≥ 10 years	19.0	13.3	21.0	14.6	21.1	25.1
Worked in the past year (yes)	69.7	57.5	63.9	71.9	75.8	79.1
Current student (yes)	89.4	91.3	90.5	88.8	88.5	88.2
Urban/rural dwelling						
Urban	80.2	76.9	79.9	74.8	82.9	86.5
Rural	19.8	23.1	20.1	25.2	17.1	13.5
Sense of community belonging						
Strong/very strong	63.6	64.5	62.1	64.9	62.3	64.2
Weak/very weak	36.4	35.5	37.9	35.1	37.7	35.8
Has a regular doctor (yes)	90.2	89.1	90.1	90.2	91.0	90.4
Mental health consultation in the past year (yes)	18.9	28.4	24.4	16.8	15.1	9.7
Usual provider of care (UPC)						
<3 visits in the past 18 months	9.8	4.8	6.2	12.0	11.7	14.3
No UPC	28.2	33.3	34.7	24.7	26.6	21.7
UPC (specialist or generalist)	62.0	61.9	59.1	63.3	61.7	64.0
Continuity of care						
< 3 visits in the past 18 months	9.8	4.8	6.2	12.0	11.7	14.3
< 50% continuity of care	28.2	33.3	34.7	24.7	26.6	21.7
50–75% continuity of care	43.0	43.9	42.2	42.7	42.8	43.3
> 75% continuity of care	19.0	18.0	16.9	20.6	18.9	20.7
Need Factors						
Has a chronic disease (one or more)	48.7	63.3	53.9	51.1	41.3	33.8
Self-perceived general health						
Poor	9.0	17.2	14.3	6.2	5.4	2.1
Fair	13.5	20.4	17.4	11.7	11.1	7.1
Good (excellent/very good)	77.4	62.4	68.3	82.1	83.5	90.8
Self-reported mental health						
Poor	2.7	4.1	4.8	1.7	2.1	1.0
Fair	8.1	11.3	12.0	6.5	6.5	4.2
Good	24.5	29.1	27.5	22.8	22.7	20.2
Very good	32.3	28.6	27.8	34.2	34.6	36.3
Excellent	32.4	26.9	27.8	34.8	34.0	38.3
Prior-year ED visits						
No visits	53.9	23.4	38.4	55.2	67.3	85.2
1–3 visits	34.1	43.7	43.0	39.7	29.8	14.2
≥ 4 visits	12.0	32.8	18.7	5.1	2.9	0.6
ADG quartile						
Q1 (lowest)	21.8	16.5	17.6	25.6	23.7	25.5
Q2	21.6	14.0	16.9	22.4	24.2	30.4
Q3	22.8	17.7	21.3	24.5	24.9	25.7
Q4 (highest)	33.8	51.8	44.2	27.5	27.2	18.4

*ED*, emergency department.

*ADG*, Aggregated Diagnosis Group; *ED*, emergency department.

**Table 2:** Weighted unadjusted, age-adjusted, and adjusted odds ratios and 95% confidence intervals according to multinomial logistic regression analysis.

	Frequent (≥ 4 visit), avoidable ED user			Frequent (≥ 4 visit), non-avoidable ED user			Infrequent (1–3 visit), avoidable ED user			Infrequent (1–3 visit), non-avoidable ED user		
	Unadjusted	Age-adjusted	Adjusted	Unadjusted	Age-adjusted	Adjusted	Unadjusted	Age-adjusted	Adjusted	Unadjusted	Age-adjusted	Adjusted
Age group												
18–30	1.00 (Ref)	-	1.00 (Ref)	1.00 (Ref)	-	1.00 (Ref)	1.00 (Ref)	-	1.00 (Ref)	1.00 (Ref)	-	1.00 (Ref)
31–40	0.63 (0.48, 0.82)	-	0.63 (0.46, 0.85)	0.60 (0.50, 0.73)	-	0.58 (0.47, 0.72)	0.82 (0.71, 0.95)	-	0.85 (0.73, 0.99)	0.83 (0.78, 0.89)	-	0.83 (0.77, 0.89)
41–50	0.51 (0.37, 0.69)	-	0.44 (0.31, 0.61)	0.68 (0.55, 0.85)	-	0.55 (0.42, 0.71)	0.66 (0.57, 0.77)	-	0.62 (0.53, 0.74)	0.78 (0.73, 0.83)	-	0.72 (0.67, 0.78)
51–60	0.86 (0.62, 1.20)	-	0.52 (0.36, 0.74)	0.69 (0.57, 0.84)	-	0.40 (0.31, 0.52)	0.86 (0.74, 1.00)	-	0.65 (0.55, 0.77)	0.79 (0.74, 0.84)	-	0.64 (0.59, 0.69)
61–74	1.50 (1.19, 1.89)	-	0.66 (0.46, 0.93)	1.00 (0.83, 1.20)	-	0.44 (0.32, 0.60)	1.14 (1.00, 1.29)	-	0.69 (0.58, 0.83)	0.96 (0.90, 1.02)	-	0.70 (0.64, 0.76)
Sex												
Female (vs male)	1.66 (1.36, 2.03)	1.66 (1.35, 2.03)	1.35 (1.09, 1.66)	1.11 (0.98, 1.27)	1.11 (0.98, 1.27)	0.93 (0.80, 1.07)	1.58 (1.43, 1.74)	1.58 (1.43, 1.74)	1.50 (1.35, 1.66)	1.00 (0.96, 1.05)	1.00 (0.96, 1.05)	0.96 (0.92, 1.01)
Education level												
Less than secondary	1.00 (Ref)	1.00 (Ref)	1.00 (Ref)	1.00 (Ref)	1.00 (Ref)	1.00 (Ref)	1.00 (Ref)	1.00 (Ref)	1.00 (Ref)	1.00 (Ref)	1.00 (Ref)	1.00 (Ref)
Secondary graduate	0.37 (0.28, 0.48)	0.39 (0.30, 0.51)	0.62 (0.47, 0.81)	0.55 (0.45, 0.66)	0.53 (0.44, 0.65)	0.83 (0.67, 1.02)	0.67 (0.57, 0.78)	0.67 (0.58, 0.78)	0.82 (0.69, 0.97)	0.76 (0.71, 0.82)	0.74 (0.69, 0.80)	0.87 (0.81, 0.94)
Some post-secondary	0.45 (0.31, 0.67)	0.49 (0.33, 0.73)	0.78 (0.53, 1.14)	0.52 (0.40, 0.68)	0.50 (0.38, 0.65)	0.78 (0.59, 1.03)	0.66 (0.54, 0.81)	0.66 (0.54, 0.81)	0.82 (0.67, 1.01)	0.80 (0.73, 0.88)	0.77 (0.70, 0.85)	0.91 (0.82, 1.01)
Post-secondary certificate	0.25 (0.20, 0.32)	0.26 (0.21, 0.33)	0.57 (0.45, 0.72)	0.38 (0.32, 0.45)	0.37 (0.31, 0.44)	0.75 (0.62, 0.90)	0.57 (0.50, 0.65)	0.57 (0.50, 0.65)	0.83 (0.71, 0.97)	0.64 (0.60, 0.68)	0.63 (0.59, 0.67)	0.81 (0.76, 0.87)
Household income												
Q1 (lowest)	5.17 (3.89, 6.86)	5.18 (3.90, 6.87)	1.91 (1.37, 2.65)	3.21 (2.58, 3.99)	3.20 (2.58, 3.99)	1.31 (0.99, 1.72)	1.40 (1.20, 1.64)	1.40 (1.20, 1.64)	1.06 (0.88, 1.29)	1.48 (1.38, 1.60)	1.48 (1.37, 1.60)	1.21 (1.11, 1.33)
Q2	2.56 (1.89, 3.48)	2.54 (1.87, 3.45)	1.39 (0.99, 1.93)	2.09 (1.67, 2.63)	2.09 (1.67, 2.63)	1.25 (0.96, 1.61)	1.31 (1.11, 1.54)	1.31 (1.11, 1.54)	1.08 (0.91, 1.30)	1.25 (1.16, 1.34)	1.25 (1.16, 1.35)	1.12 (1.03, 1.21)
Q3	2.53 (1.80, 3.55)	2.52 (1.79, 3.53)	1.50 (1.08, 2.09)	1.72 (1.37, 2.16)	1.72 (1.37, 2.16)	1.14 (0.89, 1.47)	1.40 (1.21, 1.63)	1.40 (1.21, 1.63)	1.18 (1.00, 1.39)	1.18 (1.11, 1.26)	1.18 (1.11, 1.26)	1.08 (1.01, 1.16)
Q4	1.64 (1.21, 2.24)	1.64 (1.21, 2.24)	1.24 (0.90, 1.71)	1.34 (1.08, 1.65)	1.34 (1.08, 1.65)	1.07 (0.85, 1.33)	1.16 (1.01, 1.33)	1.16 (1.01, 1.33)	1.04 (0.91, 1.20)	1.10 (1.03, 1.17)	1.10 (1.03, 1.17)	1.04 (0.97, 1.11)
Q5 (highest)	1.00 (Ref)	1.00 (Ref)	1.00 (Ref)	1.00 (Ref)	1.00 (Ref)	1.00 (Ref)	1.00 (Ref)	1.00 (Ref)	1.00 (Ref)	1.00 (Ref)	1.00 (Ref)	1.00 (Ref)
Age group												
Other vs married/ common law	1.56 (1.30, 1.89)	1.83 (1.50, 2.24)	1.08 (0.86, 1.36)	1.66 (1.45, 1.89)	1.75 (1.53, 2.01)	1.13 (0.96, 1.33)	1.19 (1.08, 1.31)	1.23 (1.11, 1.37)	1.03 (0.92, 1.15)	1.18 (1.13, 1.23)	1.18 (1.12, 1.23)	0.97 (0.92, 1.03)
Cigarette smoking status												
Non-smoker	1.00 (Ref)	1.00 (Ref)	1.00 (Ref)	1.00 (Ref)	1.00 (Ref)	1.00 (Ref)	1.00 (Ref)	1.00 (Ref)	1.00 (Ref)	1.00 (Ref)	1.00 (Ref)	1.00 (Ref)
Current smoker	2.37 (1.89, 2.96)	2.38 (1.90, 2.97)	1.32 (1.00, 1.72)	2.68 (2.29, 3.13)	2.68 (2.29, 3.13)	1.71 (1.45, 2.01)	1.68 (1.49, 1.88)	1.68 (1.49, 1.88)	1.29 (1.15, 1.46)	1.50 (1.42, 1.58)	1.50 (1.42, 1.58)	1.20 (1.14, 1.27)
Former smoker	1.64 (1.31, 2.06)	1.50 (1.19, 1.89)	1.08 (0.85, 1.38)	1.50 (1.26, 1.78)	1.51 (1.26, 1.81)	1.24 (1.02, 1.51)	1.53 (1.37, 1.72)	1.54 (1.36, 1.74)	1.26 (1.11, 1.43)	1.20 (1.13, 1.26)	1.23 (1.16, 1.30)	1.07 (1.01, 1.13)
Alcohol consumption (prior year)												
Non-drinker	1.00 (Ref)	1.00 (Ref)	1.00 (Ref)	1.00 (Ref)	1.00 (Ref)	1.00 (Ref)	1.00 (Ref)	1.00 (Ref)	1.00 (Ref)	1.00 (Ref)	1.00 (Ref)	1.00 (Ref)
Light or moderate drinker	0.36 (0.30, 0.44)	0.35 (0.29, 0.43)	0.59 (0.48, 0.73)	0.56 (0.48, 0.65)	0.56 (0.48, 0.65)	0.81 (0.69, 0.95)	0.69 (0.62, 0.76)	0.68 (0.61, 0.76)	0.78 (0.70, 0.87)	0.84 (0.80, 0.88)	0.84 (0.80, 0.88)	0.92 (0.87, 0.97)
Heavy drinker	0.73 (0.53, 1.00)	0.77 (0.56, 1.05)	0.81 (0.58, 1.13)	0.94 (0.76, 1.17)	0.95 (0.76, 1.18)	0.85 (0.68, 1.07)	0.96 (0.81, 1.13)	0.97 (0.82, 1.14)	0.89 (0.75, 1.05)	1.17 (1.09, 1.26)	1.16 (1.08, 1.25)	1.04 (0.96, 1.13)
Physical activity												
Very active	1.00 (Ref)	1.00 (Ref)	1.00 (Ref)	1.00 (Ref)	1.00 (Ref)	1.00 (Ref)	1.00 (Ref)	1.00 (Ref)	1.00 Ref)	1.00 (Ref)	1.00 (Ref)	1.00 (Ref)
Moderately active	1.20 (0.90, 1.60)	1.17 (0.87, 1.56)	1.11 (0.85, 1.45)	0.77 (0.65, 0.92)	0.77 (0.65, 0.92)	0.77 (0.64, 0.92)	1.00 (0.88, 1.14)	1.00 (0.87, 1.14)	1.00 (0.87, 1.14)	0.93 (0.87, 0.99)	0.93 (0.88, 0.99)	0.94 (0.88, 1.00)
Inactive	1.23 (0.99, 1.53)	1.19 (0.96, 1.48)	0.79 (0.63, 1.01)	1.24 (1.06, 1.44)	1.24 (1.07, 1.44)	0.94 (0.79, 1.11)	1.06 (0.95, 1.19)	1.06 (0.94, 1.19)	0.98 (0.87, 1.10)	1.00 (0.95, 1.05)	1.01 (0.95, 1.06)	0.92 (0.87, 0.97)
Life stress												
High (quite a bit, extreme) vs low	1.55 (1.28, 1.88)	1.58 (1.30, 1.92)	1.06 (0.85, 1.32)	1.66 (1.45, 1.91)	1.66 (1.45, 1.91)	1.14 (0.98, 1.33)	1.18 (1.06, 1.31)	1.18 (1.06, 1.31)	1.08 (0.96, 1.22)	1.23 (1.17, 1.30)	1.23 (1.17, 1.30)	1.10 (1.04, 1.16)
Life satisfaction												
Very high	1.00 (Ref)	1.00 (Ref)	1.00 (Ref)	1.00 (Ref)	1.00 (Ref)	1.00 (Ref)	1.00 (Ref)	1.00 (Ref)	1.00 (Ref)	1.00 (Ref)	1.00 (Ref)	1.00 (Ref)
High	1.00 (0.80, 1.26)	1.02 (0.81, 1.27)	0.81 (0.64, 1.04)	1.35 (1.14, 1.58)	1.34 (1.14, 1.58)	1.07 (0.90, 1.28)	0.94 (0.85, 1.05)	0.95 (0.85, 1.05)	0.91 (0.81, 1.02)	1.03 (0.98, 1.08)	1.03 (0.98, 1.08)	0.96 (0.91, 1.02)
Neutral	1.94 (1.38, 2.71)	1.93 (1.37, 2.70)	0.80 (0.53, 1.20)	2.55 (2.03, 3.20)	2.55 (2.03, 3.20)	1.10 (0.81, 1.48)	1.14 (0.96, 1.35)	1.14 (0.96, 1.35)	0.84 (0.69, 1.02)	1.38 (1.26, 1.51)	1.38 (1.26, 1.51)	1.02 (0.92, 1.13)
Low	4.22 (2.72, 6.55)	4.16 (2.68, 6.46)	1.00 (0.59, 1.69)	4.95 (3.58, 6.86)	4.97 (3.59, 6.88)	1.30 (0.88, 1.92)	1.24 (0.92, 1.66)	1.23 (0.92, 1.65)	0.68 (0.48, 0.95)	1.80 (1.56, 2.08)	1.81 (1.57, 2.09)	1.06 (0.91, 1.25)
Very low	5.08 (2.62, 9.87)	4.90 (2.53, 9.50)	0.70 (0.31, 1.60)	6.55 (4.06, 10.56)	6.59 (4.09, 10.64)	1.11 (0.62, 1.98)	1.48 (0.93, 2.36)	1.47 (0.92, 2.35)	0.61 (0.36, 1.03)	2.69 (2.05, 3.52)	2.72 (2.08, 3.56)	1.33 (0.94, 1.87)
Body Mass Index												
Underweight	2.60 (1.71, 3.95)	2.58 (1.70, 3.92)	1.38 (0.92, 2.06)	1.83 (1.37, 2.45)	1.83 (1.37, 2.45)	1.17 (0.88, 1.56)	1.42 (1.16, 1.75)	1.42 (1.16, 1.75)	1.08 (0.87, 1.34)	1.35 (1.21, 1.50)	1.35 (1.21, 1.51)	1.17 (1.04, 1.31)
Normal weight	1.00 (Ref)	1.00 (Ref)	1.00 (Ref)	1.00 (Ref)	1.00 (Ref)	1.00 (Ref)	1.00 (Ref)	1.00 (Ref)	1.00 (Ref)	1.00 (Ref)	1.00 (Ref)	1.00 (Ref)
Overweight	1.22 (0.98, 1.53)	1.17 (0.94, 1.45)	1.27 (1.01, 1.59)	0.86 (0.73, 1.02)	0.87 (0.74, 1.03)	0.90 (0.75, 1.08)	1.09 (0.97, 1.23)	1.10 (0.97, 1.24)	1.11 (0.99, 1.26)	1.07 (1.02, 1.13)	1.09 (1.04, 1.15)	1.08 (1.02, 1.14)
Obese	2.49 (1.96, 3.17)	2.35 (1.85, 2.99)	1.51 (1.18, 1.94)	1.62 (1.37, 1.92)	1.64 (1.37, 1.97)	1.15 (0.95, 1.40)	1.74 (1.54, 1.96)	1.75 (1.55, 1.97)	1.33 (1.17, 1.52)	1.36 (1.29, 1.44)	1.39 (1.32, 1.48)	1.18 (1.11, 1.25)
Neighbourhood-level material resources												
Q1 (least deprived)	1.00 (Ref)	1.00 (Ref)	1.00 (Ref)	1.00 (Ref)	1.00 (Ref)	1.00 (Ref)	1.00 (Ref)	1.00 (Ref)	1.00 (Ref)	1.00 (Ref)	1.00 (Ref)	1.00 (Ref)
Q2	1.52 (1.00, 2.32)	1.52 (1.00, 2.31)	1.21 (0.79, 1.86)	1.50 (1.17, 1.93)	1.50 (1.17, 1.93)	1.01 (0.79, 1.29)	1.50 (1.27, 1.77)	1.50 (1.27, 1.77)	1.14 (0.94, 1.37)	1.10 (1.03, 1.18)	1.10 (1.03, 1.18)	1.10 (1.02, 1.18)
Q3	1.82 (1.26, 2.64)	1.83 (1.26, 2.65)	1.11 (0.73, 1.69)	1.82 (1.44, 2.29)	1.82 (1.44, 2.29)	1.02 (0.78, 1.33)	1.69 (1.45, 1.97)	1.69 (1.45, 1.97)	1.18 (0.99, 1.41)	1.22 (1.14, 1.31)	1.22 (1.14, 1.31)	1.04 (0.96, 1.12)
Q4	2.75 (1.94, 3.90)	2.76 (1.95, 3.91)	1.64 (1.08, 2.50)	2.46 (1.95, 3.09)	2.46 (1.95, 3.09)	1.22 (0.96, 1.56)	1.89 (1.61, 2.21)	1.89 (1.61, 2.21)	1.40 (1.18, 1.67)	1.31 (1.22, 1.40)	1.31 (1.22, 1.40)	1.11 (1.03, 1.19)
Q5 (most deprived)	3.38 (2.42, 4.72)	3.43 (2.45, 4.80)	2.23 (1.47, 3.36)	3.05 (2.44, 3.82)	3.05 (2.45, 3.81)	1.32 (1.04, 1.67)	2.02 (1.72, 2.37)	2.03 (1.72, 2.38)	1.44 (1.21, 1.71)	1.53 (1.43, 1.65)	1.53 (1.43, 1.64)	1.15 (1.06, 1.23)
Neighbourhood-level household and dwelling												
Q1 (least unstable)	1.00 (Ref)	1.00 (Ref)	1.00 (Ref)	1.00 (Ref)	1.00 (Ref)	1.00 (Ref)	1.00 (Ref)	1.00 (Ref)	1.00 (Ref)	1.00 (Ref)	1.00 (Ref)	1.00 (Ref)
Q2	2.22 (1.52, 3.24)	2.19 (1.50, 3.19)	1.39 (0.93, 2.07)	1.40 (1.10, 1.80)	1.41 (1.10, 1.80)	1.05 (0.81, 1.36)	1.59 (1.35, 1.88)	1.59 (1.34, 1.88)	1.18 (0.99, 1.40)	1.18 (1.10, 1.27)	1.18 (1.10, 1.27)	1.05 (0.97, 1.13)
Q3	3.37 (2.33, 4.86)	3.31 (2.29, 4.78)	1.51 (1.00, 2.27)	1.62 (1.29, 2.05)	1.63 (1.29, 2.06)	0.99 (0.77, 1.28)	1.99 (1.68, 2.35)	1.99 (1.68, 2.35)	1.31 (1.09, 1.58)	1.34 (1.25, 1.44)	1.34 (1.25, 1.44)	1.12 (1.04, 1.21)
Q4	3.92 (2.71, 5.65)	3.87 (2.68, 5.59)	1.50 (1.02, 2.40)	1.79 (1.42, 2.25)	1.79 (1.42, 2.25)	0.98 (0.76, 1.28)	1.81 (1.53, 2.14)	1.81 (1.53, 2.14)	1.20 (1.00, 1.44)	1.35 (1.26, 1.45)	1.36 (1.26, 1.45)	1.09 (1.01, 1.18)
Q5 (most unstable)	2.91 (2.01, 4.21)	2.90 (2.00, 4.19)	1.05 (0.68, 1.63)	2.05 (1.61, 2.59)	2.05 (1.61, 2.60)	1.02 (0.76, 1.38)	1.52 (1.27, 1.80)	1.51 (1.27, 1.80)	1.10 (0.90, 1.34)	1.36 (1.27, 1.47)	1.36 (1.27, 1.47)	1.11 (1.02, 1.21)
Neighbourhood-level age and labour force												
Q1 (least dependency)												
Q2	1.35 (0.82, 2.23)	1.34 (0.81, 2.21)	1.21 (0.79, 1.86)	1.11 (0.87, 1.41)	1.11 (0.87, 1.42)	1.01 (0.79, 1.29)	1.26 (1.05, 1.52)	1.26 (1.05, 1.52)	1.14 (0.94, 1.37)	1.13 (1.05, 1.21)	1.13 (1.05, 1.22)	1.10 (1.02, 1.18)
Q3	1.48 (0.90, 2.43)	1.45 (0.88, 2.4)	1.11 (0.73, 1.69)	1.22 (0.95, 1.56)	1.23 (0.96, 1.58)	1.02 (0.78, 1.33)	1.55 (1.30, 1.84)	1.55 (1.31, 1.85)	1.18 (0.99, 1.41)	1.13 (1.05, 1.22)	1.14 (1.06, 1.23)	1.04 (0.96, 1.12)
Q4	2.65 (1.63, 4.31)	2.59 (1.59, 4.23)	1.64 (1.08, 2.50)	1.59 (1.27, 1.99)	1.61 (1.28, 2.01)	1.22 (0.96, 1.56)	2.01 (1.70, 2.38)	2.02 (1.71, 2.39)	1.40 (1.18, 1.67)	1.26 (1.17, 1.35)	1.27 (1.19, 1.37)	1.11 (1.03, 1.19)
Q5 (most dependency)	4.20 (2.61, 6.77)	4.05 (2.50, 6.55)	2.23 (1.47, 3.36)	1.94 (1.56, 2.40)	1.97 (1.59, 2.45)	1.32 (1.04, 1.67)	2.29 (1.95, 2.69)	2.31 (1.96, 2.72)	1.44 (1.21, 1.71)	1.39 (1.30, 1.49)	1.42 (1.32, 1.52)	1.15 (1.06, 1.23)
Racial or ethnic origin												
Visible minority vs White	0.30 (0.19, 0.46)	0.31 (0.20, 0.48)	0.58 (0.36, 0.91)	0.60 (0.47, 0.77)	0.59 (0.46, 0.76)	0.88 (0.66, 1.17)	0.34 (0.28, 0.42)	0.34 (0.28, 0.42)	0.57 (0.45, 0.71)	0.68 (0.63, 0.73)	0.67 (0.62, 0.72)	0.82 (0.75, 0.89)
First language												
Other vs English-speaking	0.61 (0.46, 0.79)	0.60 (0.46, 0.79)	1.01 (0.75, 1.36)	0.71 (0.59, 0.86)	0.71 (0.59, 0.86)	0.92 (0.74, 1.14)	0.53 (0.46, 0.60)	0.53 (0.46, 0.60)	0.82 (0.70, 0.96)	0.77 (0.72, 0.81)	0.77 (0.72, 0.81)	0.91 (0.84, 1.00)
Immigration status												
Canadian-born	1.00 (Ref)	1.00 (Ref)	1.00 (Ref)	1.00 (Ref)	1.00 (Ref)	1.00 (Ref)	1.00 (Ref)	1.00 (Ref)	1.00 (Ref)	1.00 (Ref)	1.00 (Ref)	1.00 (Ref)
Immigrant, <10 years	0.27 (0.12, 0.60)	0.29 (0.13, 0.65)	1.01 (0.41, 2.49)	0.36 (0.23, 0.57)	0.36 (0.23, 0.57)	0.73 (0.44, 1.21)	0.33 (0.24, 0.48)	0.34 (0.24, 0.49)	0.90 (0.61, 1.31)	0.61 (0.55, 0.69)	0.61 (0.54, 0.68)	0.89 (0.77, 1.02)
Immigrant, ≥10 years	0.42 (0.31, 0.57)	0.38 (0.28, 0.52)	0.70 (0.48, 1.00)	0.73 (0.59, 0.91)	0.74 (0.60, 0.91)	1.05 (0.84, 1.32)	0.47 (0.40, 0.55)	0.46 (0.39, 0.54)	0.81 (0.68, 0.98)	0.76 (0.71, 0.81)	0.77 (0.72, 0.82)	0.97 (0.89, 1.04)
Worked in the prior year												
Did not work in the past year (vs did)	2.79 (2.32, 3.36)	2.91 (2.37, 3.56)	0.91 (0.72, 1.16)	2.14 (1.87, 2.44)	2.48 (2.12, 2.90)	1.10 (0.91, 1.34)	1.48 (1.34, 1.63)	1.55 (1.38, 1.73)	1.02 (0.90, 1.17)	1.21 (1.15, 1.27)	1.29 (1.22, 1.36)	0.93 (0.87, 0.99)
Current student												
Current student (vs not)	1.41 (1.02, 1.95)	1.17 (0.84, 1.65)	1.29 (0.89, 1.86)	1.27 (1.02, 1.59)	1.35 (1.04, 1.75)	1.29 (0.99, 1.70)	1.07 (0.92, 1.23)	1.04 (0.89, 1.21)	1.00 (0.85, 1.18)	1.04 (0.97, 1.11)	1.10 (1.02, 1.18)	1.09 (1.00, 1.18)
Urban/rural dwelling												
Rural vs Urban	1.92 (1.61, 2.29)	1.87 (1.56, 2.23)	1.44 (1.18, 1.77)	1.61 (1.40, 1.84)	1.62 (1.41, 1.85)	1.50 (1.29, 1.75)	2.16 (1.95, 2.38)	2.16 (1.95, 2.38)	1.65 (1.48, 1.85)	1.32 (1.26, 1.38)	1.33 (1.27, 1.39)	1.23 (1.17, 1.30)
Sense of community belonging												
Weak/very weak vs strong/very strong	0.99 (0.79, 1.23)	1.02 (0.82, 1.27)	0.85 (0.67, 1.07)	1.09 (0.95, 1.26)	1.09 (0.95, 1.26)	0.86 (0.73, 1.00)	0.97 (0.88, 1.07)	0.97 (0.88, 1.08)	0.97 (0.87, 1.09)	1.08 (1.03, 1.14)	1.08 (1.03, 1.13)	1.02 (0.97, 1.07)
Has a regular doctor												
Yes (vs no)	0.87 (0.69, 1.09)	0.80 (0.63, 1.02)	0.73 (0.56, 0.95)	0.96 (0.78, 1.18)	0.96 (0.78, 1.18)	0.92 (0.73, 1.16)	0.97 (0.82, 1.15)	0.96 (0.81, 1.14)	0.82 (0.69, 0.97)	1.07 (0.99, 1.16)	1.09 (1.00, 1.18)	1.06 (0.97, 1.15)
Mental health consultation in the past year												
Yes (vs no)	3.71 (2.99, 4.60)	3.79 (3.06, 4.69)	1.66 (1.29, 2.13)	3.01 (2.57, 3.52)	3.01 (2.57, 3.52)	1.39 (1.16, 1.66)	1.89 (1.65, 2.16)	1.90 (1.65, 2.17)	1.26 (1.08, 1.48)	1.66 (1.57, 1.77)	1.66 (1.56, 1.77)	1.23 (1.15, 1.33)
Usual Provider of Care (UPC)												
< 3 visits	0.35 (0.25, 0.49)	0.37 (0.26, 0.52)	0.58 (0.40, 0.84)	0.47 (0.38, 0.59)	0.47 (0.38, 0.58)	0.63 (0.50, 0.79)	0.85 (0.73, 1.00)	0.86 (0.73, 1.00)	1.06 (0.90, 1.25)	0.85 (0.79, 0.91)	0.83 (0.78, 0.89)	0.92 (0.86, 0.99)
≥ 3 visits, no UPC	1.59 (1.28, 1.98)	1.60 (1.29, 1.99)	1.09 (0.89, 1.33)	1.74 (1.50, 2.01)	1.73 (1.50, 2.01)	1.30 (1.12, 1.52)	1.15 (1.03, 1.28)	1.15 (1.04, 1.29)	1.01 (0.90, 1.13)	1.27 (1.21, 1.34)	1.27 (1.21, 1.34)	1.15 (1.09, 1.21)
≥ 3 visits, specialist or generalist	1.00 (Ref)	1.00 (Ref)	1.00 (Ref)	1.00 (Ref)	1.00 (Ref)	1.00 (Ref)	1.00 (Ref)	1.00 (Ref)	1.00 (Ref)	1.00 (Ref)	1.00 (Ref)	1.00 (Ref)
Chronic diseases												
≥ 1 more vs none	3.38 (2.78, 4.10)	3.62 (2.96, 4.43)	1.73 (1.38, 2.17)	2.28 (2.00, 2.61)	2.66 (2.30, 3.07)	1.42 (1.19, 1.70)	2.04 (1.86, 2.24)	2.27 (2.05, 2.51)	1.72 (1.53, 1.93)	1.37 (1.31, 1.44)	1.50 (1.43, 1.57)	1.15 (1.09, 1.21)
Self-perceived general health												
Poor	11.89 (8.53, 16.59)	12.02 (8.54, 16.91)	2.45 (1.68, 3.58)	9.07 (7.40, 11.11)	10.18 (8.20, 12.64)	2.23 (1.72, 2.89)	3.24 (2.68, 3.91)	3.31 (2.74, 4.00)	1.84 (1.48, 2.29)	2.81 (2.52, 3.12)	2.97 (2.67, 3.31)	1.63 (1.43, 1.85)
Fair	4.19 (3.45, 5.09)	4.22 (3.46, 5.15)	1.45 (1.16, 1.83)	3.27 (2.76, 3.88)	3.54 (2.98, 4.21)	1.34 (1.09, 1.64)	1.83 (1.62, 2.08)	1.86 (1.64, 2.11)	1.18 (1.03, 1.36)	1.70 (1.59, 1.83)	1.77 (1.65, 1.91)	1.22 (1.13, 1.33)
Good (excellent/ very good)	1.00 (Ref)	1.00 (Ref)	1.00 (Ref)	1.00 (Ref)	1.00 (Ref)	1.00 (Ref)	1.00 (Ref)	1.00 (Ref)	1.00 (Ref)	1.00 (Ref)	1.00 (Ref)	1.00 (Ref)
Self-reported mental health												
Poor/fair vs Good/excellent/very good)	2.36 (1.94, 2.86)	2.34 (1.93, 2.83)	1.08 (0.84, 1.39)	2.33 (2.01, 2.70)	2.33 (2.02, 2.70)	1.15 (0.95, 1.40)	1.32 (1.19, 1.46)	1.32 (1.19, 1.46)	1.02 (0.90, 1.15)	1.34 (1.28, 1.41)	1.34 (1.28, 1.41)	1.02 (0.96, 1.08)
Prior-year ED visits												
No ED visits	1.00 (Ref)	1.00 (Ref)	1.00 (Ref)	1.00 (Ref)	1.00 (Ref)	1.00 (Ref)	1.00 (Ref)	1.00 (Ref)	1.00 (Ref)	1.00 (Ref)	1.00 (Ref)	1.00 (Ref)
1–3 ED visits	11.19 (9.11, 13.73)	11.22 (9.14, 13.77)	7.15 (5.83, 8.78)	6.71 (5.77, 7.81)	6.71 (5.77, 7.81)	4.54 (3.88, 5.32)	4.31 (3.91, 4.75)	4.31 (3.91, 4.76)	3.33 (3.01, 3.70)	2.66 (2.53, 2.79)	2.66 (2.53, 2.79)	2.19 (2.08, 2.30)
≥ 4 ED visits	212.97 (162.59, 278.96)	214.55 (163.82, 280.99)	82.13 (61.60, 109.50)	73.97 (60.66, 90.21)	74.00 (60.69, 90.24)	31.17 (25.11, 38.69)	14.02 (11.11, 17.69)	14.04 (11.13, 17.72)	8.45 (6.57, 10.86)	6.61 (5.74, 7.62)	6.60 (5.73, 7.61)	4.34 (3.76, 4.99)
ADG quartile												
Q1 (lowest)	1.00 (Ref)	1.00 (Ref)	1.00 (Ref)	1.00 (Ref)	1.00 (Ref)	1.00 (Ref)	1.00 (Ref)	1.00 (Ref)	1.00 (Ref)	1.00 (Ref)	1.00 (Ref)	1.00 (Ref)
Q2	0.71 (0.52, 0.97)	0.73 (0.53, 0.99)	1.04 (0.77, 1.43)	0.80 (0.64, 1.01)	0.84 (0.67, 1.06)	1.03 (0.80, 1.31)	0.73 (0.64, 0.84)	0.74 (0.65, 0.85)	0.87 (0.75, 1.00)	0.86 (0.81, 0.91)	0.88 (0.83, 0.93)	0.94 (0.88, 1.00)
Q3	1.06 (0.8, 1.42)	1.16 (0.86, 1.57)	0.98 (0.71, 1.34)	1.20 (0.99, 1.45)	1.42 (1.17, 1.72)	1.15 (0.93, 1.41)	0.95 (0.83, 1.09)	0.99 (0.86, 1.14)	0.88 (0.77, 1.02)	1.04 (0.98, 1.11)	1.13 (1.06, 1.21)	1.04 (0.98, 1.11)
Q4 (highest)	4.35 (3.40, 5.56)	5.10 (3.92, 6.64)	1.70 (1.28, 2.26)	3.47 (2.92, 4.14)	4.700 (3.85, 5.73)	1.82 (1.45, 2.27)	1.49 (1.32, 1.69)	1.60 (1.40, 1.82)	0.94 (0.82, 1.09)	1.59 (1.49, 1.69)	1.83 (1.71, 1.96)	1.28 (1.19, 1.38)

Reference group = Individuals with no ED use in the year following CCHS interview (No ED usage).

Age-adjusted = multivariable multinomial logistic regression model adjusting for age, in years, as a continuous variable.

Adjusted = multivariable multinomial logistic regression model including all covariates listed in Table 2.

*ED*, emergency department.
