# Supplementary material for: Sociodemographic and Health Behaviour of Frequent, Avoidable Emergency Department Users in Ontario, Canada: A Population-based Descriptive Study
Source: West J Emerg Med. 2025 Oct 21;26(6):1622–39. doi: 10.5811/westjem.46551 (PMC12698170; doi:10.5811/westjem.46551)
Supplement: Supplementary file 1 [file wjem-26-1622-s001.docx]

**Additional File 1**

A summary of ICD-9 and ICD-10 codes used for identifying ambulatory care sensitive conditions using Canadian Institute for Health Information (CIHI) administrative data. (Ambulatory Care Sensitive Conditions, Canadian Institute of Health Information https://www.cihi.ca/en/indicators/ambulatory-care-sensitive-conditions)

| **Condition** | **Codes** |
| --- | --- |
| Grand mal status and other epileptic convulsions | ICD-9/9-CM: 345  ICD-10-CA: G40, G41 |
| Chronic lower respiratory diseases (except asthma) | ICD-9/9-CM: 491, 492, 494, 496  ICD-10-CA: J41, J41, J43, J44, J47  MRDx of acute lower respiratory infection, only when a secondary diagnosis of JFF in ICD-10-CA or ICD-9/9-CM is also present  ICD-9/9-CM: 466, 480-486, 487.0  ICD-10-CA: J10.0, J11.0, J12-J16, J18, J20, J21, J22 |
| Asthma | ICD-9-9-CM: 493  ICD-10-CA: J45 |
| Diabetes | ICD-9: 250.0, 250.1, 250.2, 250.7 ICD-9-CM: 250.0, 250.1, 250.2, 250.8 ICD-10-CA: E10.0, E10.1, E10.63, E10.64, E10.9, E11.0, E11.1, E11.63, E11.64, E11.9, E13.0, E13.1, E13.63, E13.64, E13.9, E14.0, E14.1, E14.63, E14.64, E14.9 |
| Heart failure and pulmonary edema | ICD-9/9-CM: 428, 518.4 ICD-10-CA: J81 (MRDx), I50 (MRDx), I50 as diagnosis type (1) when I11 is MRDx |
| Hypertension | ICD-9/9-CM: 401.0, 401.9, 402.0, 402.1, 402.9 ICD-10-CA: I10 (MRDx), I11 as MRDx when I50 as diagnosis type (1) is not present |
| Angina | ICD-9: 411, 413 ICD-9-CM: 411.1, 411.8, 413 ICD-10-CA: I20, I23.82, I24.0, I24.8, I24.9  Excluding cases with cardiac procedures. List of procedure codes for exclusion:  CCP: 47, 480–483, 489.1, 489.9, 492–495, 497, 498 ICD-9-CM: 336, 35, 36, 373, 375, 377, 378, 379.4–379.8 CCI: 1.HA.58., 1.HA.80., 1.HA.87., 1.HB.53., 1.HB.54., 1.HB.55., 1.HB.87., 1.HD.53., 1.HD.54., 1.HD.55., 1.HH.59., 1.HH.71., 1.HJ.76., 1.HJ.82., 1.HM.57., 1.HM.78., 1.HM.80., 1.HN.71., 1.HN.80., 1.HN.87., 1.HP.76., 1.HP.78., 1.HP.80., 1.HP.82., 1.HP.83., 1.HP.87., 1.HR.71., 1.HR.80., 1.HR.84., 1.HR.87., 1.HS.80., 1.HS.90., 1.HT.80., 1.HT.89., 1.HT.90., 1.HU.80., 1.HU.90., 1.HV.80., 1.HV.90., 1.HW.78., 1.HW.79., 1.HX.71., 1.HX.78., 1.HX.79., 1.HX.80., 1.HX.83., 1.HX.86., 1.HX.87., 1.HY.85., 1.HZ.53 rubric (except 1.HZ.53.LA-KP), 1.HZ.54., 1.HZ.55 rubric (except 1.HZ.55.LA-KP), 1.HZ.56., 1.HZ.57., 1.HZ.59., 1.HZ.80., 1.HZ.85., 1.HZ.87., 1.IF.83., 1.IJ.50., 1.IJ.54.GQ-AZ, 1.IJ.55., 1.IJ.57., 1.IJ.76., 1.IJ.80., 1.IJ.86., 1.IK.50., 1.IK.57., 1.IK.80., 1.IK.87., 1.IN.84., 1.LA.84., 1.LC.84., 1.LD.84., 1.YY.54.LA-NJ, 1.YY.54.LA-FS, 1.YY.54.LA-NM, 1.YY.54.LA-FR, 1.YY.54.LA-FU |

Where: ICD = International Classification of Diseases; ACSC = ambulatory care sensitive condition; MRx = most responsible diagnosis

A summary of the inclusion and exclusion criteria for the SNC definition for avoidable Emergency Department visits used in this study. (Ontario District Health Councils Local Health System Monitoring Technical Working Group. Access, equity & integration indicators for local health system monitoring in Ontario [Internet]. Toronto: Ontario District Health Councils; 2004)

| **Inclusion Criteria** | **Exclusion Criteria** |
| --- | --- |
| Unscheduled visits to emergency rooms (not scheduled follow-up appointments) | People less than one year of age or age 75 and older |
| ICD-10-CA codes: A740, B309, H100, H101, H102, H103, H104, H105, H108, H109, H130, H131, H132, H133, N300, N301, N302, N303, N304, N308, N309, N330, N390, H650, H651, H652, H653, H654, H659, H660, H661, H662, H663, H664, H669, H670, H671, H678, J00, J010, J011, J012, J013, J014, J018, J019, J028, J029, J038, J039, J040, J041, J060, J068, J069, J310, J311, J312, J320, J321, J322, J323, J324, J328, J329, J350, J351, J352, J353, J358, J359, J399  ICD-9 codes:, 030.9, 372.30, 372.14, 364.24, 381.00, 381.01, 381.02, 381.023, 381.04, 381.4, 382.9, 382.00,382.01, 382.02, 382.03, 384.0, 384.1, 384.8, 384.9, 460, 461.0, 461.1, 461.2, 461.3, 461.8, 461.9, 034.8, 462, 463, 464.00, 464.10, 472.0, 472.8, 472.1, 473.0, 473.1, 473.2, 473.3, 473.8, 473.9, 474.00, 474.10, 474.80, 595.0, 595.1, 595.2, 595.3, 595.89, 595.9, 593.2, 599.0, 711.0, 743.34 | Emergency visits resulting in an inpatient admission |
| Canadian Emergency Department Triage and Acuity Scale (CTAS) levels 4 and 5 (less urgent, non-urgent) | Out-of-province patients |

Where: ICD = International Classification of Diseases; SNC = sentinel nonurgent condition
