# Supplementary material for: Sociodemographic and Health Behaviour of Frequent, Avoidable Emergency Department Users in Ontario, Canada: A Population-based Descriptive Study
Source: West J Emerg Med. 2025 Oct 21;26(6):1622–39. doi: 10.5811/westjem.46551 (PMC12698170; doi:10.5811/westjem.46551)
Supplement: Supplementary file 2 [file wjem-26-1622-s002.docx]

**Additional File 2**

Summary of covariates included in this study, scale, and source of data informing the variable.

| **Variable** | **Scale** | **Source** |
| --- | --- | --- |
| **Predisposing Factors** | | |
| Age | Categorical:  18 to 30 years  31 to 40 years  41 to 50 years  51 to 60 years  61 to 74 years | CCHS interview |
| Sex | Binary: male, female | CCHS interview |
| Education | Categorical:  Less than secondary  Secondary graduate  Some post-secondary  Post-secondary certificate | CCHS interview |
| Income | Household income, quintiles | CCHS interview |
| Marital status | Binary: married or common-law, other | CCHS interview |
| Cigarette smoking status | Categorical:  Non-smoker  Light smoker (<1 pack per day)  Heavy smoker (>1 pack per day)  Former smoker | CCHS interview |
| Alcohol consumption status | Categorical:  Non-drinker  Light or moderate drinker  Heavy drinker | CCHS interview |
| Physical activity level | Categorical:  Very active  Light or moderately active  Inactive | CCHS interview |
| Life stress | Binary: low (a bit, not very, none), high (quite a bit, extreme) | CCHS interview |
| Life satisfaction | Categorical:  Very high  High  Normal  Low  Very low | CCHS interview |
| Body Mass Index | Categorical:  Underweight (<18.5kg/m^2^)  Normal (18.5 – 24.9kg/m^2^)  Overweight (25 – 29.9 kg/m^2^)  Obese (30 kg/m^2^+) | CCHS interview |
| **Enabling Factors** | | |
| Neighbourhood-level material resources | Quintiles | Ontario Marginalization Index |
| Neighbourhood-level household and dwelling | Quintiles | Ontario Marginalization Index |
| Neighbourhood-level age and labour force | Quintiles | Ontario Marginalization Index |
| Racial or ethnic origin | Binary: white, visible minority | CCHS interview |
| Immigration status | Categorical:  Canadian-born  Immigrant <10 years  Immigrant 10+ years | CCHS interview |
| Worked in the past year | Binary: yes, no | CCHS interview |
| Currently a student | Binary: yes, no | CCHS interview |
| Urban or rural dwelling | Binary: urban, rural | CCHS interview |
| Sense of community belonging | Binary: strong/very strong, weak/very weak | CCHS interview |
| Regular family doctor | Binary: yes, no | CCHS interview |
| Mental health consultation in the past year | Binary: yes, no | CCHS interview |
| Usual Provider of Care | Categorical:  <3 visits in the past 18 months  No usual provider of care (<50% of ambulatory care visits with the same provider)  Usual provider of care GP or specialist | OHIP/CIHI-NACRS |
| Continuity of care | Categorical:  <3 visits in the past 18 months  <50% of ambulatory care visits with the same provider  50-75% of ambulatory care visits with the same provider  >75% of visits with the same provider | OHIP/CIHI-NACRS |
| **Need Factors** | | |
| Chronic disease/comorbidities | Binary: none, one or more | ICES-derived algorithms |
| Self-perceived physical health | Categorical:  Poor  Fair  Excellent, very good, good | CCHS |
| Self-reported mental health | Categorical:  Poor  Fair  Good  Very good  Excellent | CCHS |
| Past-year Emergency Department utilization | Categorical:  No visits  1-3 visits  4+ visits | CIHI-NACRS |
| Aggregated Diagnosis Group | Quartiles | OHIP/DAD |

Where: CCHS = Canadian Community Health Survey; OHIP = Ontario Health Insurance Plan; CIHI-NACRS = Canadian Institute for Health Information National Ambulatory Care Reporting System; DAD = Discharge Abstract Database
